# Adipose-derived stem cell sheets accelerate bone healing in rat femoral defects

**DOI:** 10.1371/journal.pone.0214488

**Published:** 2019-03-28

**Authors:** Yasuhisa Yoshida, Hidenori Matsubara, Xiang Fang, Katsuhiro Hayashi, Issei Nomura, Shuhei Ugaji, Tomo Hamada, Hiroyuki Tsuchiya

**Affiliations:** Department of Orthopaedic Surgery, Graduate School of Medical Science, Kanazawa University, Kanazawa, Japan; Università degli Studi della Campania, ITALY

## Abstract

In the present study, we investigated whether both adipose-derived stem cell (ADSC) and osteogenic-induced ADSC sheets could promote bone healing in a rat distal femoral metaphysis bone defect model. A through-hole defect of 1 mm diameter was drilled into each distal femur of 12 week old rats. Forty-five rats were randomly assigned to three groups: (1) control group; (2) ADSC sheet group; or (3) osteogenic-induced ADSC sheet group. We evaluated each group by analysis of computerized tomography scans every week after the surgery, histological analysis, and DiI labeling (a method of membrane staining for post implant cell tracing). Radiological and histological evaluations showed that a part of the hole persisted in the control group at four weeks after surgery, whereas the hole was restored almost completely by new bone formation in both sheet groups. The mean value of bone density (in Houndsfield units) for the bone defect area was significantly higher in both sheet groups than that in the control group (*p* = 0.05) at four weeks postoperative. A large number of osteocalcin positive osteoblasts were observed at the area of bone defect, especially in the osteogenic-induced ADCS sheet group. DiI labeling in the newly formed bone showed that each sheet had differentiated into bone tissue at four weeks after surgery. The ADSC and the osteogenic-induced ADSC sheets promoted significantly quicker bone healing in the bone defect. Moreover, the osteogenic-induced ADSC sheet may be more advantageous for bone healing than the ADSC sheet because of the higher number of osteocalcin positive osteoblasts via the transplantation.

## Introduction

In the field of orthopedics, large bone defects are often encountered after comminuted fracture, bone infection, osteonecrosis, or tumor resection. In order to reconstruct such bone defects, a variety of methods have been employed including autogenous bone grafting [1], vascularized fibular transplantation [2], distraction osteogenesis [3], and artificial bone grafting using materials derived from calcium phosphate [4] or hydroxyapatite [5]. In particular, autogenous bone constitutes a superior material for reconstruction but exhibits some disadvantages such as limited amounts of harvestable material and concerns regarding donor sites [1,6]. Allografting is less common because of supply difficulties owing to social reasons (especially in Japan) and concerns about the risk of infection [7]. Finally, artificial bone grafts may not yield complete repair, as the strength and osteoinduction afforded by a single application may be insufficient.

Mesenchymal stem cells (MSCs) are capable of differentiating into adipocyte, osteocyte, chondrocyte, and other mesodermal lineages [8], and can also differentiate into other lineages such as neurons [9] and hepatocytes [10]. Therefore, MSCs serve as an important component in regenerative medicine as their pluripotency supports the reconstruction of various tissues. For example, bone marrow stem cells (BMSCs) have been used in bone regeneration research for at least four decades [11] and their validity has been examined from various perspectives. However, BMSCs have some major limitations, as the isolation procedure is invasive for donors and patients, and often only low numbers of stem cells are obtained following the isolation. In comparison, adipose tissue has recently been attracting attention as an alternative source of MSCs because the tissue collection is relatively simple and less invasive in humans [12,13]. Notably, since the first report of preadipocytes contained in adipose tissue by Green and Meuth in 1974 [14], numerous research studies have been conducted. In particular, Zuk et al. reported in 2001 that human adipose tissue contains multipotent cells [13] termed adipose-derived stem/stromal cells (ADSCs) [15].

We previously established and reported a method for readily forming ADSC sheets by adding ascorbic acid in vitro and demonstrated that the fabricated sheets have the capacity to be induced to differentiate into osteoblasts [16]. Several additional reports on MSC sheets or similar tissues-engineered constructs [17–21] have found that such cell sheets have the advantage of being able to be transplanted without a carrier and can serve as a scaffold at the transplant site. However, to our knowledge there have been no attempts to use ADSC sheets for the treatment of bone defects without a scaffold, such as in artificial bone grafts. We therefore hypothesized that the ADCS sheets could accelerate and enhance the bone regeneration and bone reconstruction without scaffolds. Accordingly, in the present study we established drilled-hole bone defects in the rat distal femoral metaphysis, which were filled with a transplantation of an ADSC sheet or an osteogenic-induced ADSC sheet. The aim of the present study was to ascertain whether the transplanted ADSC sheets could promote bone healing in such a bone defect model.

## Materials and methods

### Study design

The experiments were conducted with the approval of the Institute for Experimental Animals, Kanazawa University Advanced Science Research Center. A total of 45, 7 week old female Fisher 344 rats (Japan SLC Co., Shizuoka, Japan) were purchased and used as donors and recipients. All rats received surgery (described in further detail below) to remove adipose tissue for the supply of ADSCs or a sham surgery, and were randomly assigned to three groups. The control group (15 rats) underwent surgery to make a drilled-hole defect of 1 mm diameter in the left distal femur, after which the wound was immediately closed; the ADSC sheet group (15 rats) received implantation of an ADSC sheet into the drilled-hole; and the osteogenic-induced ADSC sheet group (15 rats) received implantation of an osteogenic-induced ADSC sheet into the drilled-hole. Each group received computerized tomography (CT) scans every week after the surgery. At 2 and 4 weeks after surgery, the rats were euthanized and the left femur was resected with the surrounding tissues. The specimens from these rats were evaluated using histologic analysis and cell labeling.

### Preparation of ADSCs

ADSCs were prepared by modifying previously reported methods [16,22,23]. Adipose tissue was obtained from the right inguinal region of a 7-week-old rat and readily immediately with phosphate buffered saline (PBS; Wako Pure Chemical Corp., Osaka, Japan). The tissue was cut into small pieces for 5 min using scissors. Collagenase (Wako Pure Chemical Corp.) was solubilized in PBS to 0.12% final concentration in 20 mL and used for dissolving adipose tissue during 45 min in a water bath at 37 °C. During the period of digestion, the mixture was stirred at intervals of 15 min. After the reaction was completed, 20 mL standard medium (Dulbecco’s modified Eagle’s medium (Wako Pure Chemical Corp.) containing 10% fetal bovine serum (FBS; Nichirei Biosciences Inc., Tokyo, Japan), and 1% Penicillin-Streptomycin solution (P/S; Wako Pure Chemical Corp.)) was immediately added to buffer the collagenase activity prior to filtering the resultant solution. The filtrate yields were centrifuged at 170 × *g* for 5 min at 25 °C, and the supernatant fluid was suctioned carefully and removed. The resulting stromal vascular fraction was cultured in standard medium in an incubator at 37 °C. After a 3 h incubation, the culture dishes were washed with PBS three times to remove the unattached cells. Only ADSCs were considered to remain on the bottom surface. The ADSCs were cultured with standard medium and were subcultured when the cells covered 80% of the area of the culture dishes. ADSCs at passage 3 were used for this study.

## Preparation of ADSC sheets

We have previously reported the method for formation of ADSC sheets [16]. Briefly, ADSCs at passage 3 were cultured in 6 cm dishes (TPP Techno Plastic Products AG, Schaffhausen, Switzerland) with standard medium until they reached a state of overconfluence. Then, the ADSC sheet medium (Dulbecco’s modified Eagle’s medium containing 10% FBS, 1% P/S, and ascorbic acid) was added to the culture dishes for generating ADSC sheets. ADSC sheet medium was changed every three days. The ADSCs were cultured in an incubator at 37 °C throughout the incubation period. ADSC sheets were formed within 1 week.

### Preparation of osteogenic-induced ADSC sheets

We have also previously reported the method for osteogenic-induced ADSC sheet preparation [16]. Osteogenic medium (minimum essential medium α (Wako Pure Chemical Corp.) containing 10% FBS, 1% P/S, 50 μM ascorbic acid, 10 mM β-glycerophosphate, and 0.1 μM dexamethasone) was used for osteogenic induction of ADSC sheets. Osteogenic medium was added into the culture dishes of the ADSC sheets for 1 week. The osteogenic medium was changed gently every three days during the induction period. The ADSC sheets were cultured in an incubator at 37 °C during the entire incubation period.

### Osteogenic ability test of the osteogenic-induced ADSC sheets

After 1 week osteogenic induction, samples of the sheets were picked out randomly to test their osteogenic ability and activity [16,23–25]. The osteogenic ability of the osteogenic-induced ADSC sheets was tested using a 1-step NBT/BCIP plus suppressor solution (Thermo Fisher Scientific Inc., Tokyo, Japan). The ADSC sheets were rinsed with PBS three times and fixed in 4% paraformaldehyde phosphate buffer (Wako Pure Chemical Corp.) for 5 min at room temperature then washed with deionized water. Next, the fixed cells were incubated with 1-step NBT/BCIP plus suppressor solution. After 30 min incubation at 37 °C, the ADSC sheets were washed with deionized water and observed both grossly and using a light microscope.

### Surgical procedure and sheet transplantation

**Fig 1:**
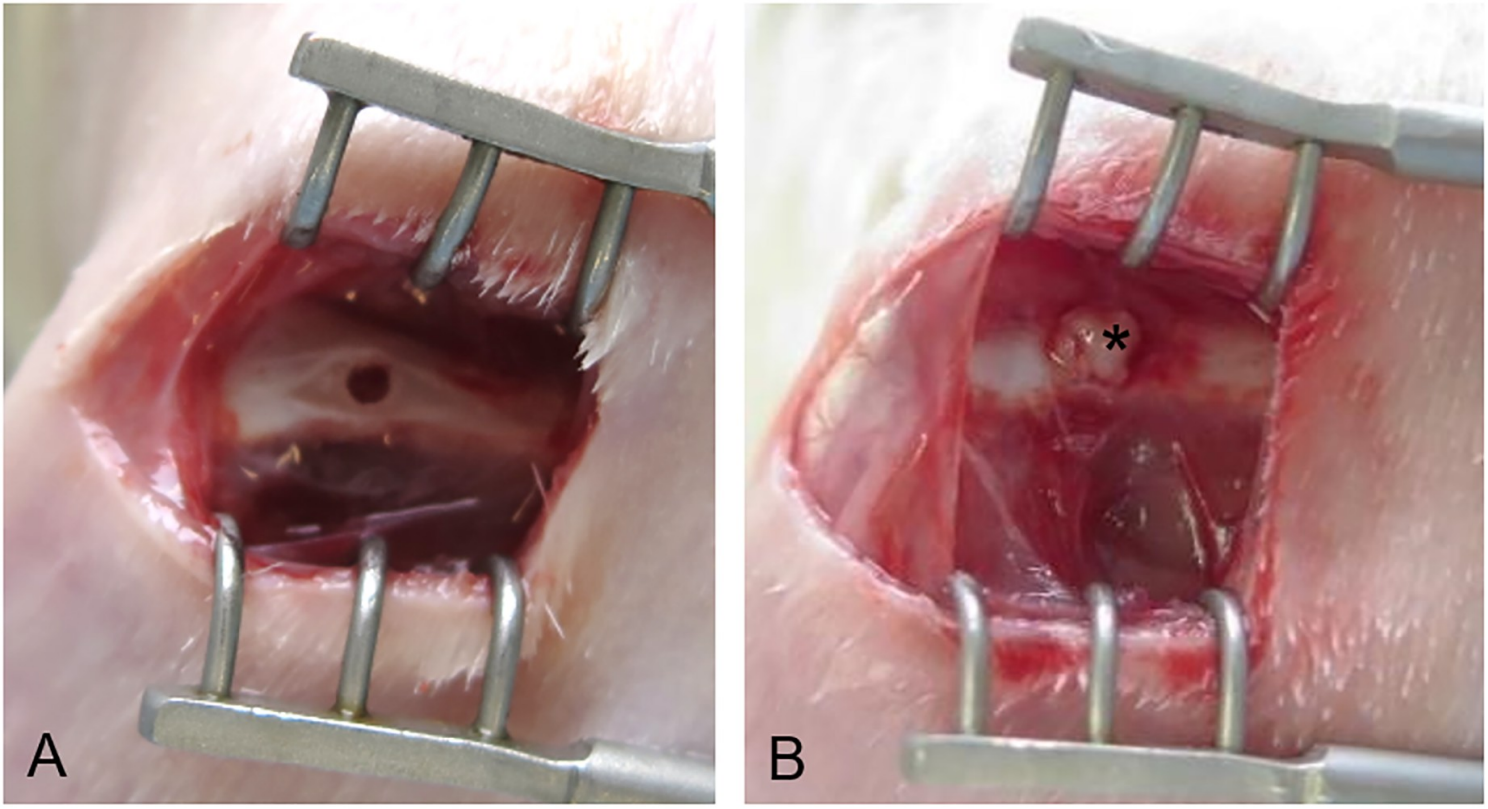
ADSC sheet transplantation into the drilled hole at the left distal femur. (A) Asterisk indicates an ADSC sheet wedged into the hole. A through-hole defect of 1 mm diameter was drilled using a Kirschner wire. (B) The hole was filled with the equivalent of the sheet of a 6 cm dish.

The surgery was performed on 12 week old rats in reference to previously reported methods [26,27] using sterile techniques according to the Guide for the Care and Use of Laboratory Animals, 8th edition published by the National Research Council (revised in 2010). Anesthesia was achieved by intraperitoneal injection with 48 mg kg^−1^ sodium pentobarbital (Somnopentyl; Kyoritsuseiyaku Corp., Tokyo, Japan). Each rat was placed in a lateral position and a lateral longitudinal incision was made in the left femur skin, followed by separation of the quadriceps femoris and hamstrings. A through-hole defect of 1 mm diameter was drilled into the left distal femur using a Kirschner wire of 1 mm in diameter. In the control group, the wound was immediately closed. In each sheet group, all of the animals were autografted and the hole was filled with the equivalent of the sheet of a 6 cm dish (Fig 1A and 1B). The fascia and skin were closed with absorbable sutures. The rats were not limited with regard to postanesthetic movement in their cages. The rearing cage that approved by the Animal Care and Use Committee was an environment suitable for usual rearing of rats. All rats remained in good health during the period of experimentation. There was no local inflammatory response around the surgical wound or implantation site. At 2 and 4 weeks after surgery, the rats were euthanized for the specimens by intraperitoneal injection of excess sodium pentobarbital.

### Radiographic evaluation

X-ray microtomograph images of the left femur were taken using a CT apparatus for experimental animals (Model LaTheta LCT-200; Hitachi-Aloka, Tokyo, Japan) [28] at every week until four weeks after the surgery (n = 10 for each group). The DICOM data that were obtained were utilized for quantitative assessments using the DICOM viewer software, Onis version 2.5 (DigitalCore Co., Ltd., Tokyo, Japan). A coronal section image via the axis of the drill-hole of the left femur was made with the drill-hole area set as the region of interest, and the bone density in terms of Houndsfield units (HU) was calculated automatically by the Onis software.

### Histological examination

In order to quantitatively assess new bone formation, bone marrow cells, and osteoblasts, we performed histological examinations using hematoxylin and eosin (H/E) staining, and osteocalcin immunostaining. In each group, two rats were euthanized at two and four weeks after surgery. The operated femurs were fixed in 4% paraformaldehyde in PBS, pH 7.4. The specimens were decalcified in 10% ethylenediaminetetraacetic acid solution, embedded in paraffin, sectioned in the coronal plane via the axis of the drill-hole, and stained with H/E. Each consecutive sectioned tissue was stained using the bone glaprotein antibody reagent for osteocalcin (Bone Gla-protein Antibody (Cat. No. 250483); Abbiotec, Inc., San Diego, CA, USA). To analyze the contents of bone marrow cells from H/E staining or osteocalcin positive osteoblasts from osteocalcin immunostaining, two sections that were obtained from specimens at two weeks after surgery from each group were selected. From each section, the objective cells were estimated in five randomly chosen fields of 10,000 μm^2^ within the area of bone defect in accordance with a previously reported method [29]. Among 10 areas (total area: 100,000 μm^2^) per group, the measurements were performed as semiquantitative assessment of bone marrow cells or osteocalcin positive osteoblasts using an image analysis program (Image-J 1.32 for Windows; National Institutes of Health, Bethesda, MD, USA) [30]. The average number of each objective cell type per 10,000 μm^2^ was compared between each group.

### DiI labeling

To establish survival potential and location of the implanted cells, ADSCs and osteogenic-induced ADSCs were labeled with DiI (Vybrant1 DiI Cell Labeling Solution; Life Technologies, Carlsbad, CA, USA) and transplanted following the sheet formation of each cell [26]. DiI is a lipophilic membrane stain that diffuses to stain the target cells and has long-term stability; this red fluorescent dye is often used as a tracer for various cells in the host tissue. At two and four weeks after transplantation of the labeled sheet, a frozen section of the coronal plane via the axis of the drill-hole was prepared. The sections were stained with H/E after determining the location of the labeled cells (n = 6 per each sheet group).

### Statistical analysis

Statistical analysis was performed using the Kruskal-Wallis H-test as multiple comparisons among groups and the Mann-Whitney U-test with Bonferroni correction as the post hoc test. A *p*-value of < 0.05 was considered significant in accordance with literature standards.

## Results

### Preparation of ADSC sheets and osteogenic-induced ADSC sheets

**Fig 2:**
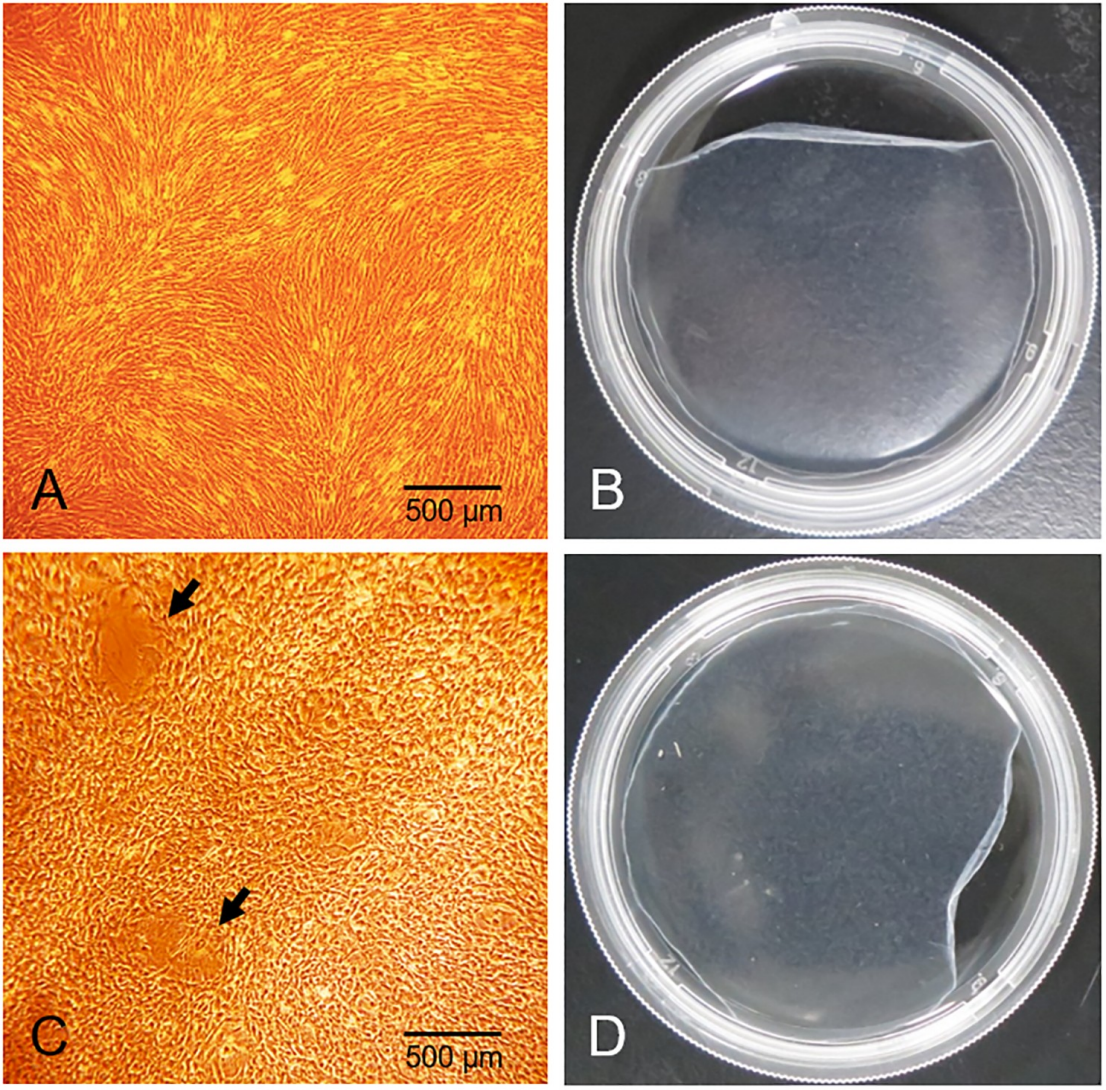
Macroscopic observation of ADSC and osteogenic-induced ADSC sheets. (A) 40X light microscopy view and (B) macroscopic appearance of an ADSC sheet (6 cm dish). (C) 40X light microscopy view and (D) macroscopic appearance of an osteogenic-induced ADSC sheet (6 cm dish). Although there were no marked changes between the macroscopic appearances (B, D), it could be observed that confluent ADSCs had an overall spindle shape (A) and osteogenic-induced ADSCs were partially round shaped (C; black arrows) in each microscopy image.

ADSC sheets were prepared after 1 week by culture in ADSC sheet medium; osteogenic-induced ADSC sheets were prepared from the ADSC sheets cultured for an additional week with the osteogenic medium. The sheets could be lifted and applied by tweezers or other tools because of their reliable mechanical strength. The confluent ADSCs were arranged tightly and retained their spindle shape; in comparison, several osteogenic-induced ADSCs were respectively island-shaped. Microscope images of each sheet are shown in Fig 2.

### Osteogenic ability test of the osteogenic-induced ADSC sheets

**Fig 3:**
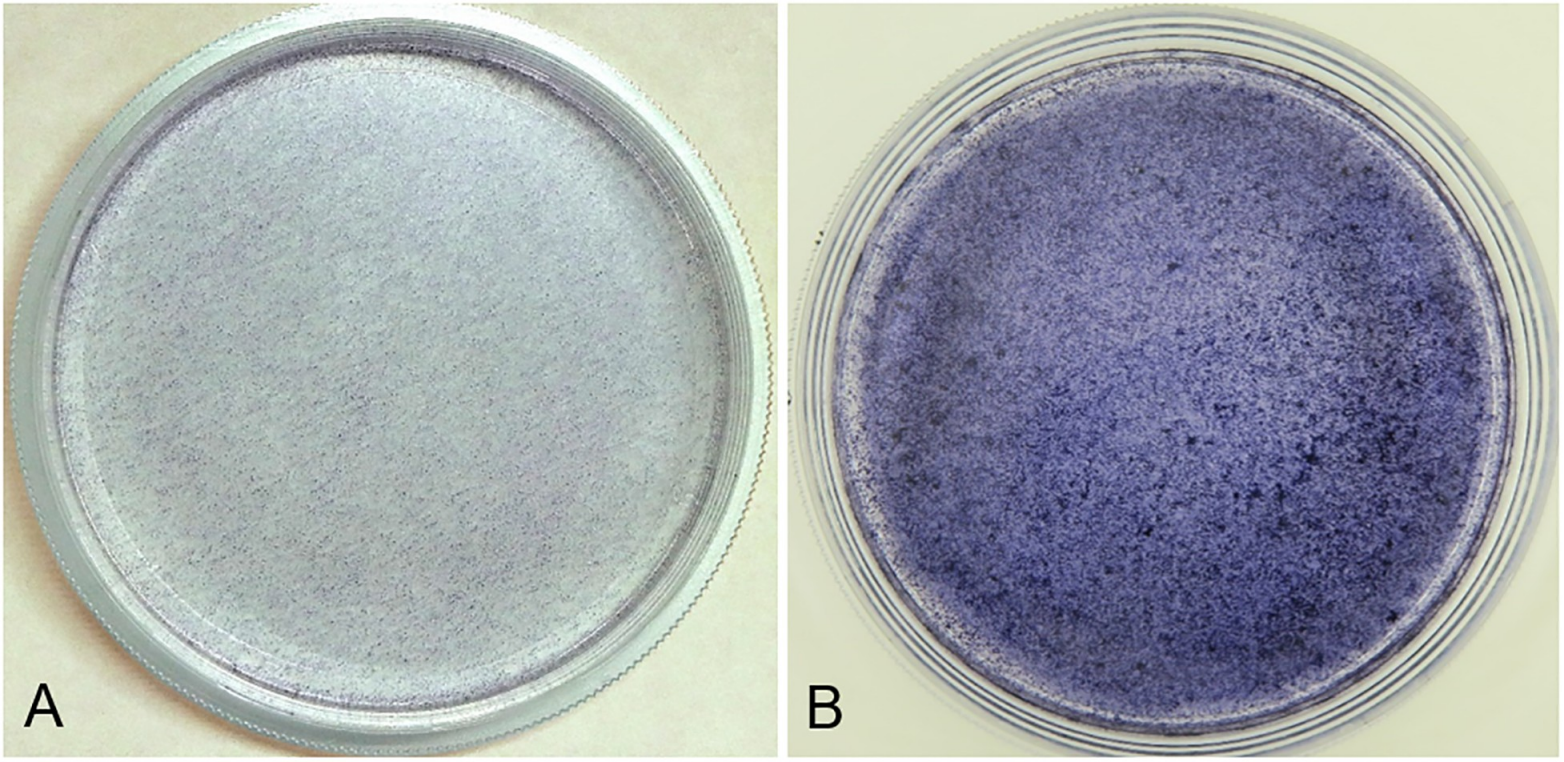
Macroscopic appearance of positive alkaline phosphatase stained ADSC and osteogenic-induced ADSC sheets. (A) A weak positive alkaline phosphatase stained ADSC sheet and (B) a strong alkaline phosphatase stained osteogenic-induced ADSC sheet (6 cm dish).

ADSCs of the ADSC sheets were induced to differentiate into osteoblasts by culturing for 1 week in osteogenic medium. Compared to weak positive stained ADSC sheet, strong positive alkaline phosphatase staining confirmed the osteogenic differentiation as the sheets were stained purple (Fig 3).

### Radiographic evaluation

**Fig 4:**
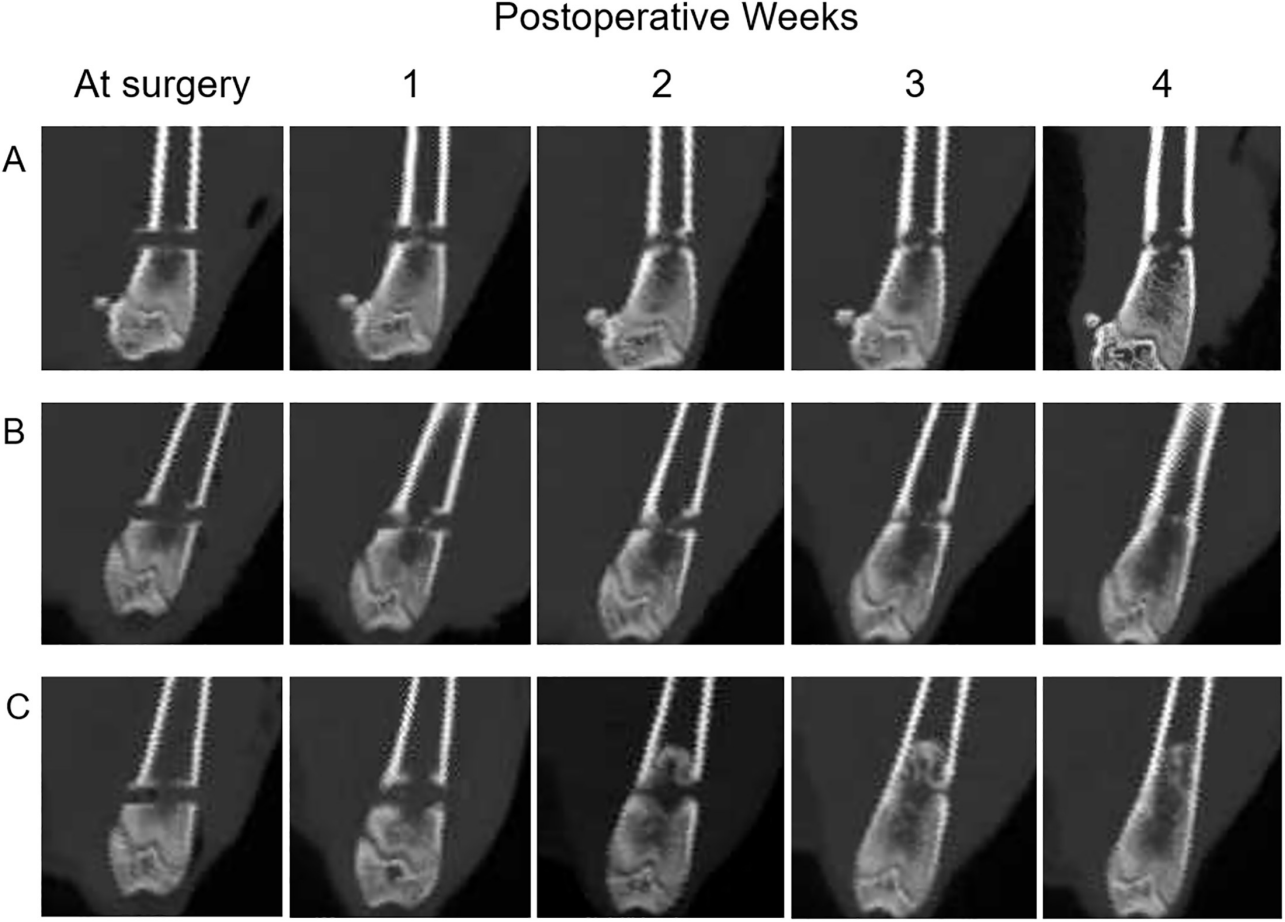
Representative CT images of rat femurs in the different groups. Femurs from (A) control, (B) ADSC sheet, and (C) osteogenic-induced ADSC sheet groups showing new bone formation at two, three, and four weeks after surgery. Bone healing appeared to occur earlier in both of the sheet groups than in the control group.

From approximately 2 weeks after the surgery, new bone formation was gradually confirmed in each group. A part of the hole persisted in the control group at four weeks after the surgery whereas the hole was restored almost completely by new bone formation in both sheet groups around the same time (Fig 4).

**Fig 5:**
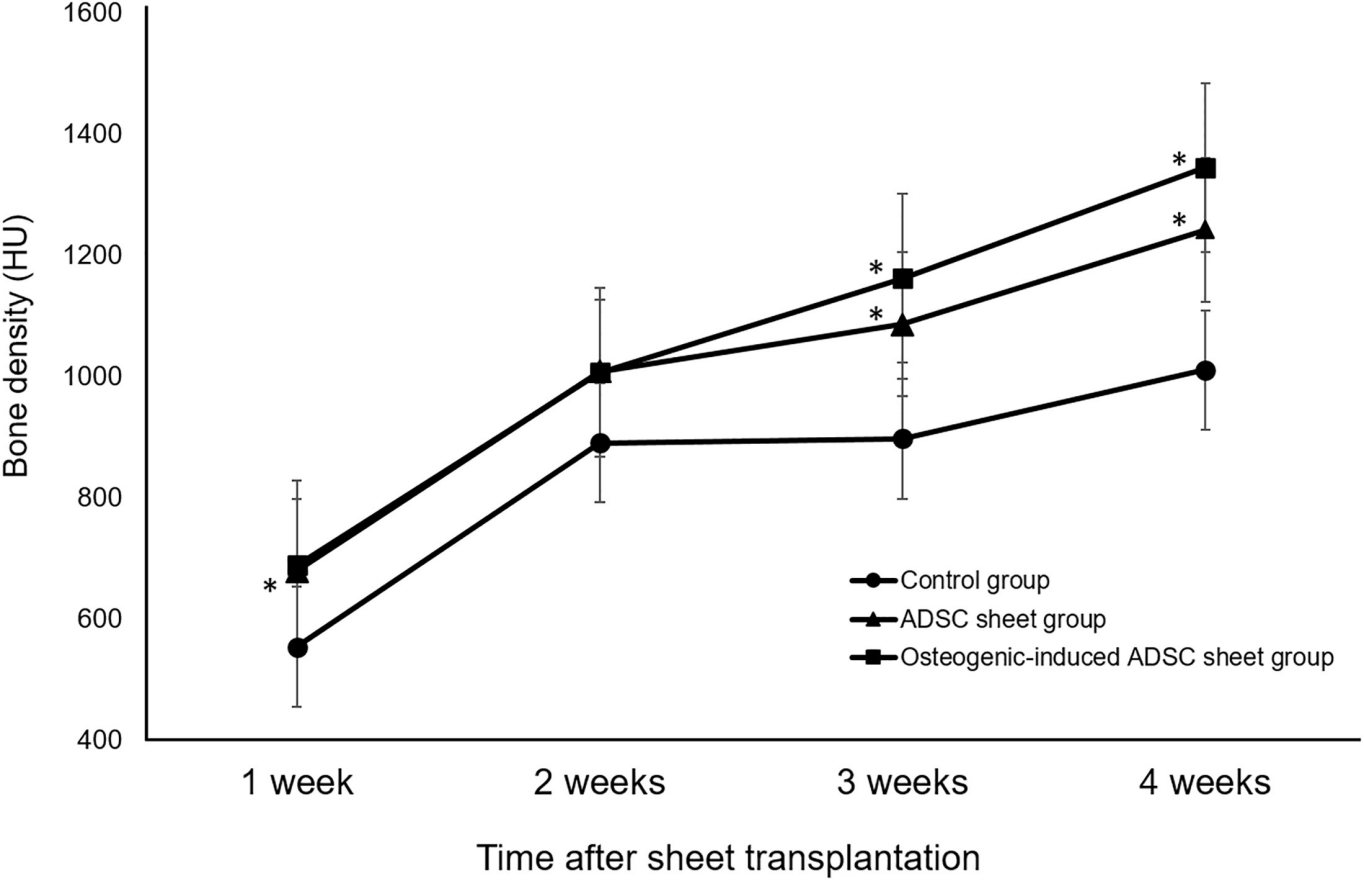
Time course of bone density changes after surgery. The ADSC sheet group bone density (in HU) was significantly greater at one, three, and four weeks than that in the control group (*p < 0.05). The bone density of the osteogenic-induced ADSC sheet group was significantly greater at three and four weeks than that in the control group (*p < 0.05).

The mean value of bone density (in Houndsfield units) for the bone defect area showed a significantly difference in each group at three and four weeks (*p* = 0.001) (Fig 5). The ADSC sheet group bone density (in HU) was significantly greater at one, three, and four weeks than that in the control group (mean ± SD, week 1, *p* = 0.035, Cohen’s d = 1.21, 95% confidence interval [CI] ± 55.777; week 3, *p* = 0.011, Cohen’s d = 1.70, 95% CI ± 72.455; week 4, *p* = 0.002, Cohen’s d = 2.19, 95% CI ± 48.299). Similarly, the bone density of the osteogenic-induced ADSC sheet group was significantly greater at three and four weeks than that in the control group (mean ± SD, week 3, *p* = 0.004, Cohen’s d = 1.94, 95% CI ± 100.929; week 4, *p* = 0.015, Cohen’s d = 1.86, 95% CI ± 136.021). At four weeks after the transplantation, the bone density had a tendency to be higher in the osteogenic-induced ADSC sheet group than in the ADSC sheet group, although there was no significant difference between the sheet groups (*p* = 0.11).

### Histological examination

**Fig 6:**
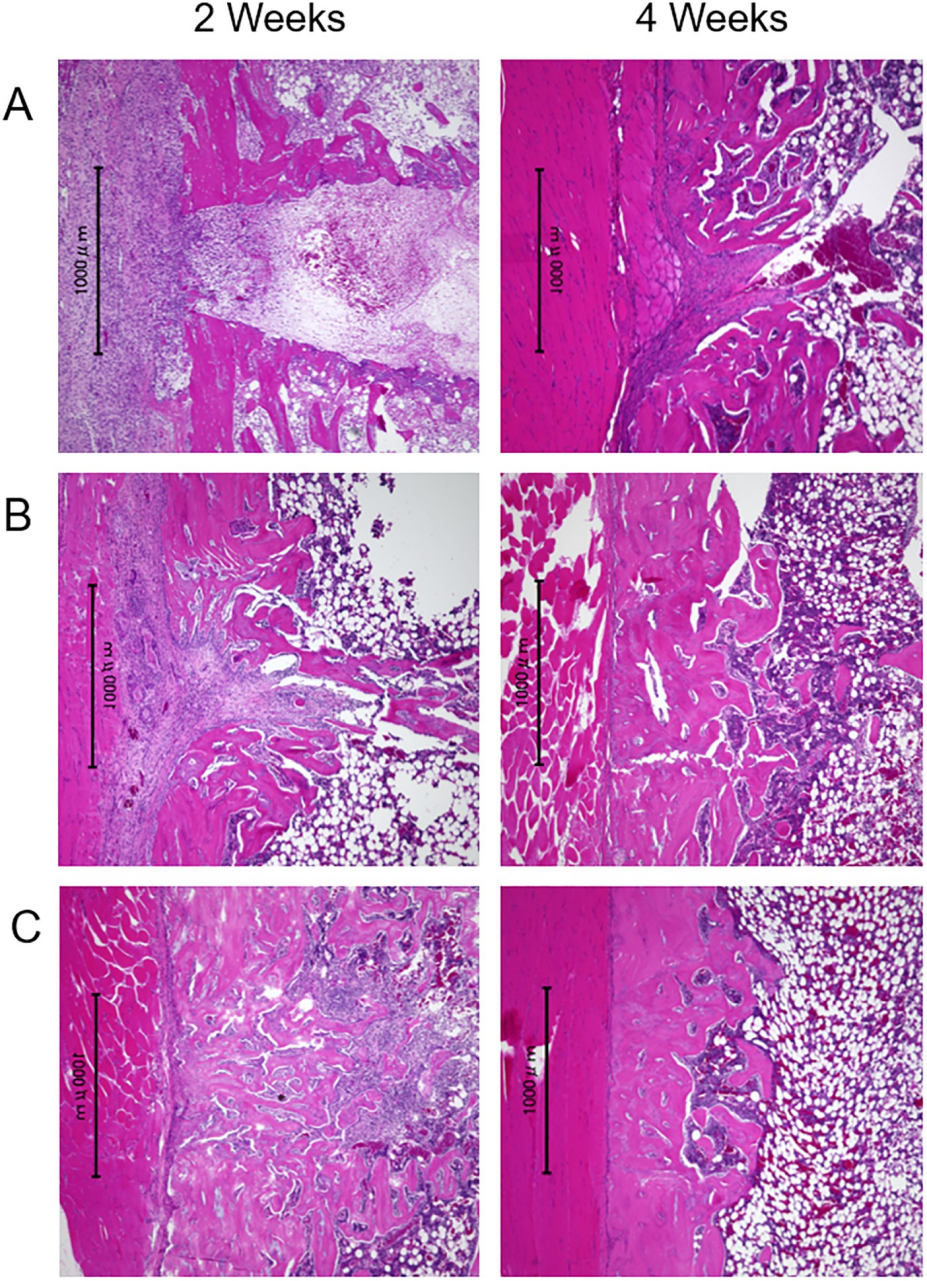
Photomicrographs of each group prepared two and four weeks after surgery. (A) Control group; (B) ADSC sheet group; and (C) osteogenic-induced ADSC sheet group; H/E stain; original magnification, X40. The control group image shows that a part of the hole persisted at four weeks after surgery. In both of the sheet groups, the hole was restored almost completely by new bone cortex at four weeks after transplantation. Moreover, the remodeling of osseous structure progressed by four weeks after the transplantation, especially in the osteogenic-induced ADSC sheet group.

**Fig 7:**
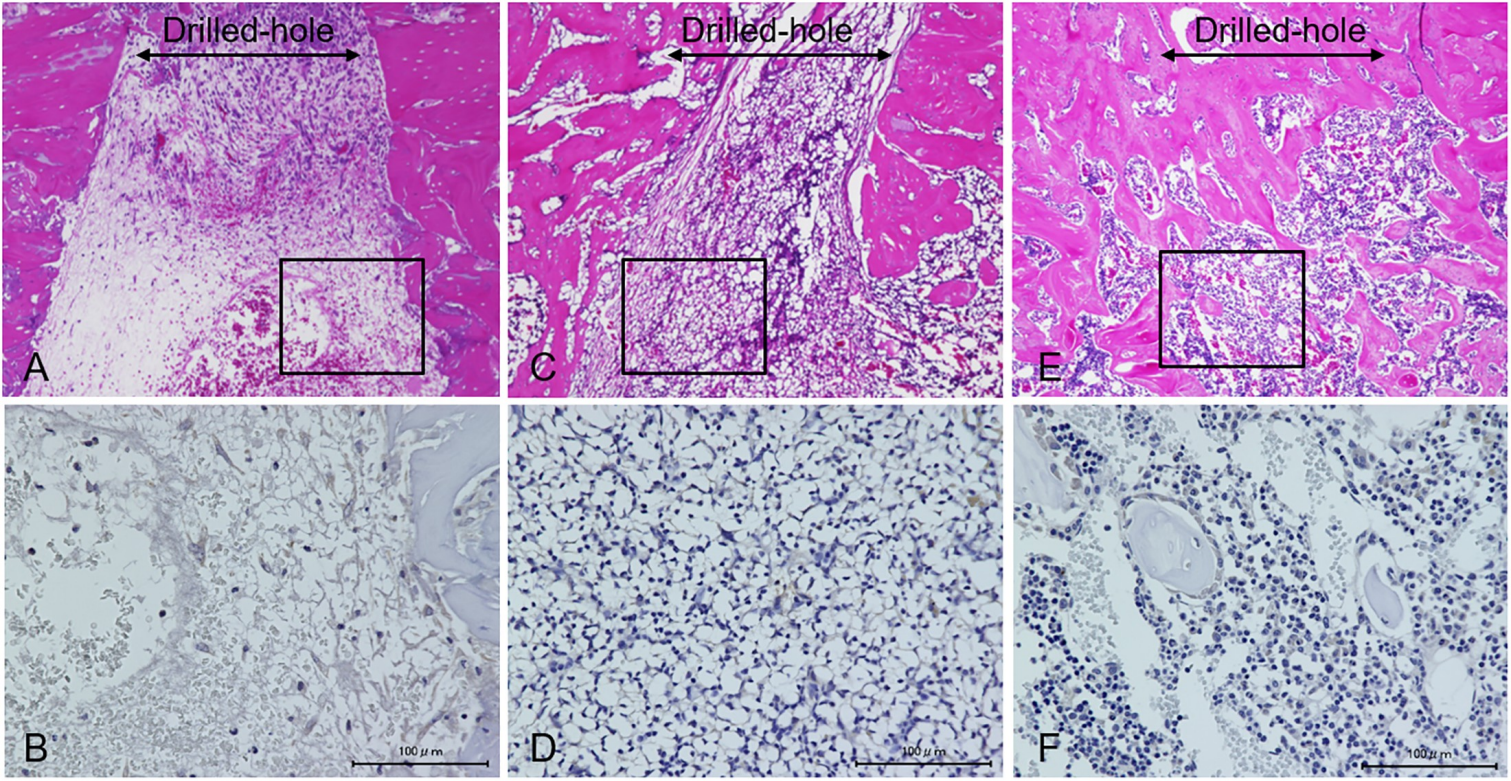
Photomicrographs at two weeks after the surgery. (A, B) Control group; (C, D) ADSC sheet group; and (E, F) osteogenic-induced ADSC sheet group. Photomicrographs of the upper section are from H/E staining (original magnification X20), and the lower section are from osteocalcin immunostaining (original magnification X400). In the osteogenic-induced ADSC sheet group, a large number of osteocalcin positive osteoblasts are distributed around new bone within the area of the bone defect.

**Fig 8:**
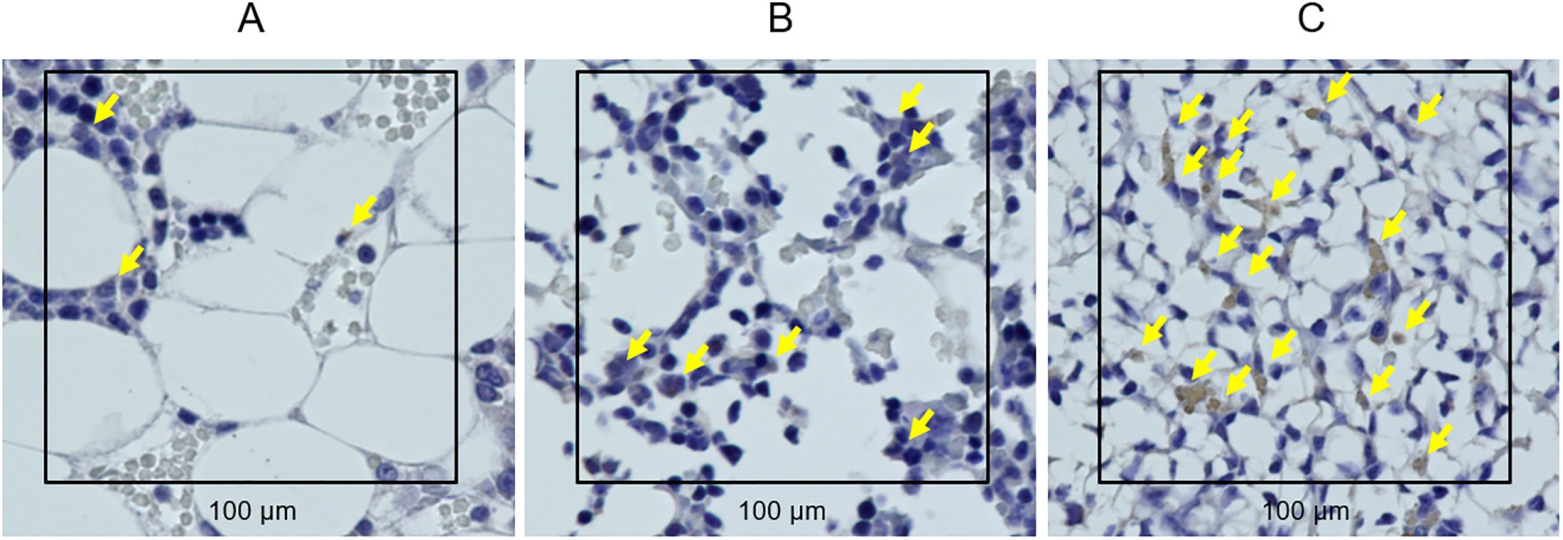
Osteocalcin immunostaining. Two weeks after the surgery of the (A) control group; (B) ADSC sheet group; and (C) osteogenic-induced ADSC sheet group; original magnification X400. To analyze the contents of osteocalcin positive osteoblasts, we randomly chose fields of 10,000 μm^2^ within the area of bone defect (black square). Osteocalcin positive osteoblasts are indicated by yellow arrows.

Fig 6 shows the photomicrographs (H/E staining) at two and four weeks after the surgery in each group. A part of the hole persisted in the control group at four weeks after the surgery; conversely, in both sheet groups, it can be observed that bone healing occurred by two weeks after the transplantation and the hole was restored almost completely by new bone formation at four weeks after transplantation. In addition, the remodeling of osseous structure progressed four weeks after the transplantation especially in the osteogenic-induced ADSC sheet group. The photomicrographs of osteocalcin immunostaining shows the existence of a large number of osteocalcin positive cells in the drill hole defect at two weeks after the transplantation of the osteogenic-induced ADSC sheet (Figs 7 and 8).

**Fig 9:**
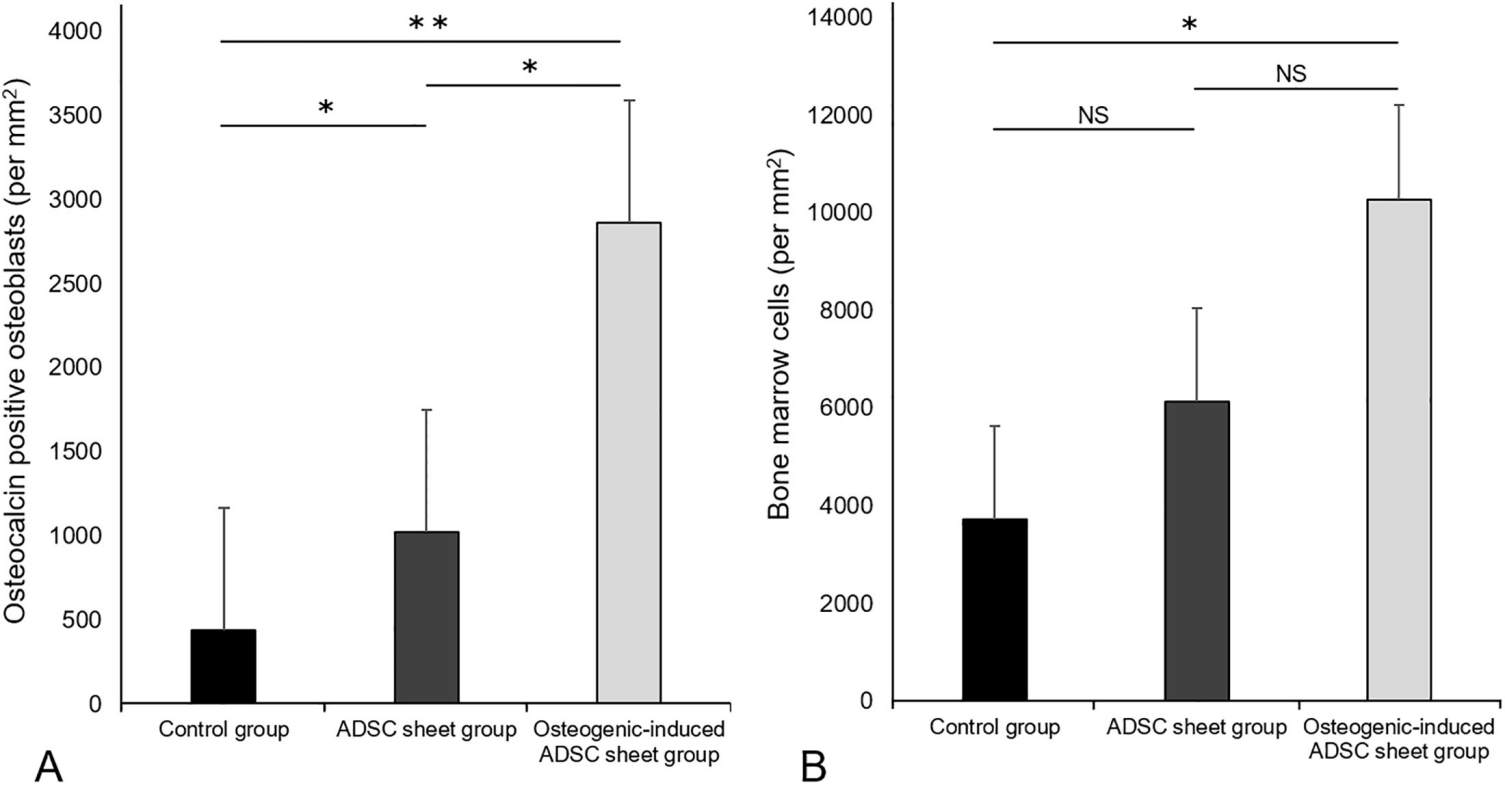
Contents of osteocalcin positive cell types in each group. Osteoblasts (A) and bone marrow cells (B) in each group are shown. Values represent the means ± SD (n = 10). *p < 0.05, **p < 0.001. NS, not significant.

The contents of osteocalcin positive osteoblasts (Fig 9A) and bone marrow cells (Fig 9B) in the osteogenic-induced ADSC sheet group were significantly higher than those of the control group at two weeks after transplantation, with the former also being significantly higher than that in the ADSC sheet group as well.

### DiI labeling

**Fig 10:**
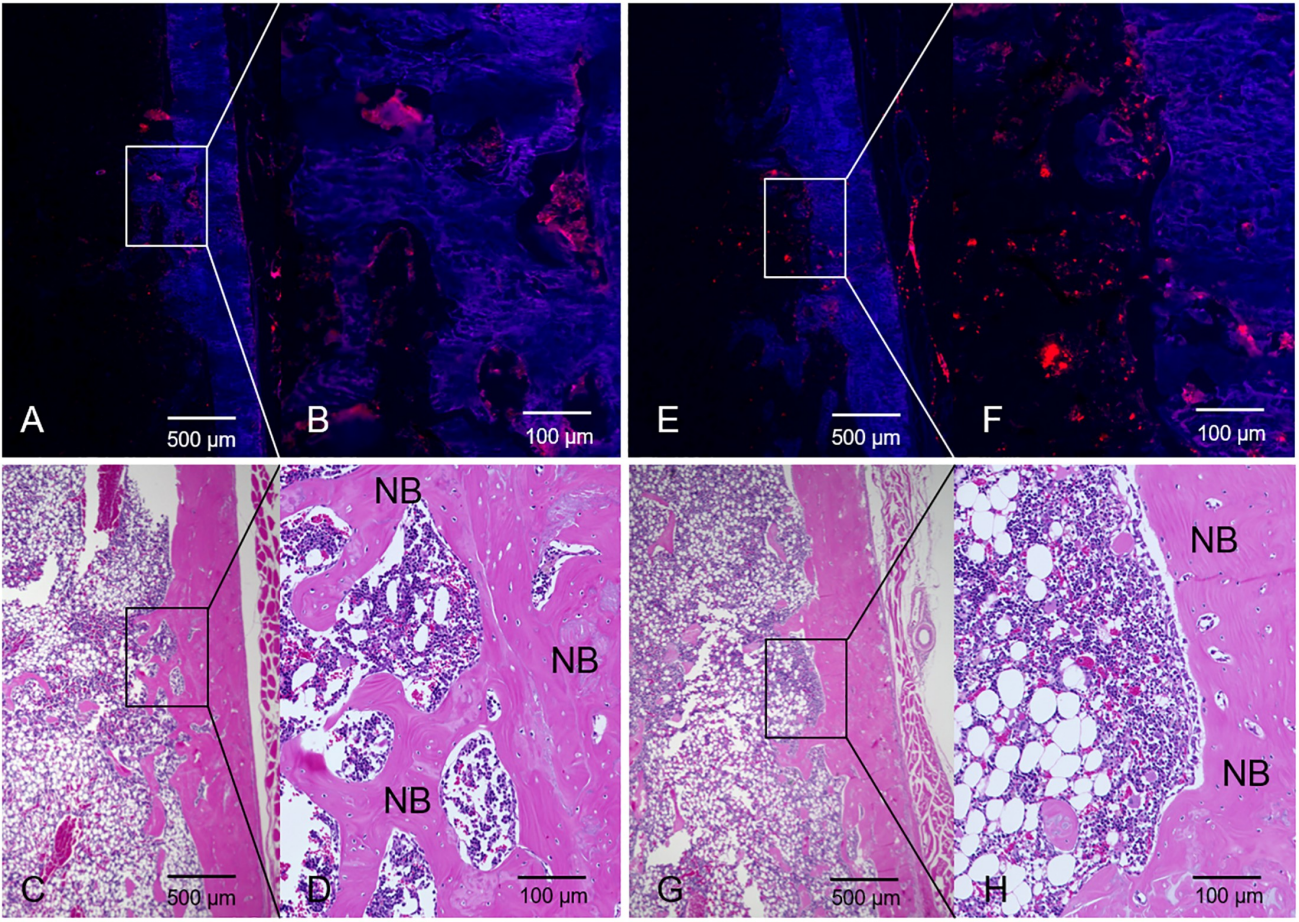
DiI labeling. At four weeks after transplantation of an ADSC sheet labeled with DiI dye, frozen sections were prepared (A, B) and stained with H/E (C, D); original magnification X20, X200. DiI (E, F) and H/E (G, H) staining at four weeks after transplantation of an osteogenic-induced ADSC sheet. Some cells survived and were focally distributed around the new bone cortex. NB = new bone.

DiI labeling evaluation suggested that the transplanted cells had survived until four weeks posttransplantation. DiI positive (red) areas were detected in a drill hole defect, which had replaced new bone tissue during the 4 weeks after transplantation of each cell sheet. The ADSC sheets and osteogenic-induced ADSC sheets that were transplanted survived up to four weeks after transplantation, with some cells being focally distributed in the new bone (Fig 10).

## Discussion

The in vivo osteogenic ability of ADSC sheets has been demonstrated in an animal model of bone defects for the first time. Bone healing was achieved at four weeks postoperatively following transplantation of both ADSC sheets and osteogenic-induced ADSC sheets, with the latter able to further advance the repair of the bone defect.

Within the interests of regenerative medicine, a variety of different stem cells have been studied to date including pluripotent stem cells such as embryonic stem cells and induced pluripotent stem cells, and tissue stem cells such as MSCs. In particular, in the field of bone regeneration, previous studies have mainly utilized several MSCs including BMSCs, ADSCs, periodontal ligament progenitor cells, and periosteum-derived stem cells [31]. Among these MSCs, although there are more reports on bone defect repair using BMSCs than ADSCs [19,20,32], the use of ADSCs offers some obvious advantage. First, adipose tissue can be harvested with less invasion [12,13]; e.g., there was almost no blood loss when we harvested adipose tissue from the inguinal region of the rat. In contrast, harvesting bone marrow requires well trained personnel, and blood loss and pain cannot be avoided. Second, the isolation of BMSCs frequently yields low numbers of stem cells. Moreover, procedures for promoting BMSC proliferation while maintaining their differentiation ability have not been established [33]. In comparison, numerous studies have reported that ADSCs may present similar or even higher regenerative ability compared to BMSCs, exhibiting (1) a faster rate of cell proliferation; (2) secretion of more regenerative factors; and (3) a higher ability of immune suppression [34–36]. The use of ADSCs is also not complicated by ethical and technical issues as in the case of embryonic stem cells or induced pluripotent stem cells. For these reasons, ADSCs are considered to represent suitable material for osteogenesis, with several studies toward this end having already been reported [36–41]. On the other hand, recently there are some studies raising concerns regarding their use in regenerative medicine, as ADSCs may promote tumor progression [42].

For clinical repair, it is necessary to consider three key factors: cells, scaffolds, and cytokines, which have been described as the “triangle concept” for in vivo osteogenesis [43]. To date, animal experiments for repairing bone defects have employed only scaffold implantation, cell implantation, or combined scaffold-cell implantation. Among the scaffolds, the implants bearing similarity to native bone composition including β-tricalcium phosphate, hydroxyapatite, and type I collagen have been reported to be effective in the repair of bone defects [44–48]. Moreover, the implantation of a scaffold-cell combination has demonstrated better osteogenic capacity that that obtained when the scaffolds were co-transplanted with MSCs [20,37,39,49,50]. In transplanting MSCs, important factors include whether the cells can be transported into the surgical site and whether the implanted cells can be functionally engrafted therein. It is considered that separated MSCs cannot themselves be fixed and remain easily within the bone defect area. Therefore, the MSCs need to be applied together with certain carriers or scaffolds. In order to transplant and retain the cells effectively, several studies have employed animal-originated type I collagen as a carrier of ADSCs for implantation into the surgical site [26,51]. Based on prior research [16,18,52], ADSC sheets and osteogenic-induced ADSC sheets were used in the present experiments, as these can be obtained by the addition of quite common and safe materials such as ascorbic acid [16], and the sheet fabrication process has been reported to increase the collagen protein-secretion of the ADSCs and osteogenic–induced ADSCs. Notably, it has been reported that the collagen protein mainly consists of type I collagen; thus, collagen is considered to represent a good natural carrier and scaffold for the sheets. In addition, the type I collagen can also facilitate inducing the ADSCs to differentiate into osteoblasts, as shown in our previous in vitro study [16]. The type I collagen contained in the ADSC sheets and osteogenic-induced ADSC sheets thus plays a role as a carrier and scaffold for the ADSCs and osteogenic-induced ADSCs, such that each sheet that was transplanted into the holes in the present experiment was fixed in place by the type I collagen network. The collagen matrix network therefore makes it possible to implant ADSCs efficiently, with the implanted ADSCs being readily able to flow out from the implanted sites without separate fixation. In particular, in the present study, each of ADSC and osteogenic-induced ADSC sheets was wedged into the hole without ripping at the time of surgery; subsequently, a large number of the transplanted cells were observed upon histological examination, with the results of DiI labeling evaluation verifying that the transplanted cells had survived until four weeks posttransplantation. However, the time required to prepare the ADSCs sheet might represent a limitation in the clinical setting. Nevertheless, the present study is the first to show the therapeutic potential of ADSC and osteogenic-induced ADSC sheets that could be used without scaffolds in animal models of bony defects of the long bone, with both the cell number and the osteogenic inducer leading to the enhancement of bone defect repair.

In the present study, we further examined whether the osteogenic-induced ADSC sheet might accelerate bone healing more effectively than that afforded by the ADSC sheet. On histological examination, it was observed that the remodeling of osseous structure had progressed to a greater degree in the osteogenic-induced ADSC sheet group, and osteocalcin immunostaining showed that the content of osteocalcin positive osteoblasts in the osteogenic-induced ADSC sheet was significantly higher than that in the ADSC sheet. However, on radiographic evaluation, the sheets did not markedly differ in their appearance on CT images; moreover, although the mean value of bone density for the bone defect area had a tendency to be higher in the osteogenic-induced ADSC sheet group than in the ADSC sheet group, there was no significant difference between the sheet groups. Although good results have been reported in studies using osteogenic-differentiated BMSC sheets for repair in a rat bone defect model, comparison of the osteogenic-differentiated BMSC sheets and non-osteogenic BMSC sheets was not performed in these studies [19,20]. Chondrogenically differentiated BMSCs have also been reported as beneficial for promoting endochondral ossification in a massive rat femur defect [32]. In addition, factors other than osteogenic differentiation may contribute to accelerate bone healing, as ADSCs are primitive stem cells compared to osteogenic-induced ADSCs and can secrete more cytokines to improve tissue repair [15,53–55]. Further study is needed to investigate the details of these processes.

The present study had some limitations. First, we did not verify the optimal quantity of the sheets to effect bone repair. For example, excess implanted cell sheet might obstruct bone remodeling, whereas too little might leave a dead space or cause other problems. Second, we did not verify the abilities of ADSC or osteogenic-induced ADSC sheets in a bone fracture model, which is relevant as more cases of bone fracture are encountered in clinical practice than of bone defects. Third, we did not demonstrate the efficacy of the sheets in a larger animal or a more massive bone defect model.

Our study showed that autogenous ADSC and osteogenic-induced ADSC sheets promoted bone healing in bone defects in vivo. Because of the efficacy of each autografting method, we hope to continue to develop these new cell-based studies for imminent clinical application. Our subsequent research will investigate the relationships between the secreted cytokines and the ADSC and osteogenic-induced ADSC sheets. The bone defect repair process using ADSC and osteogenic-induced ADSC sheets also needs to be understood at the cytokine molecular level [34,51]. Moreover, the biomechanical status of the surgical sites also needs to be evaluated in future studies. The present findings provide a foundation to eventually facilitate bone formation after trauma or tumor resection in human bones.

## Conclusion

The present study demonstrated that ADSC and osteogenic-induced ADSC sheets could advance the repair of bone defects. Although several issues remain to be investigated including the cytokine molecular mechanism, the application of ADSC sheets and osteogenic-induced ADSC sheets appears to represent a promising option for bone defect reconstruction.

## Supporting information

S1 File Details of the results. Time course of bone density changes, and contents of osteocalcin positive cell types.
(XLSX)
